# Commercialized Blood-, Urinary- and Tissue-Based Biomarker Tests for Prostate Cancer Diagnosis and Prognosis

**DOI:** 10.3390/cancers12123790

**Published:** 2020-12-16

**Authors:** Wieke C. H. Visser, Hans de Jong, Willem J. G. Melchers, Peter F. A. Mulders, Jack A. Schalken

**Affiliations:** 1 Department of Product Development, MDxHealth BV, 6534 AT Nijmegen, The Netherlands; hans.dejong@mdxhealth.com (H.d.J.); Willem.Melchers@radboudumc.nl (W.J.G.M.); 2 Department of Medical Microbiology, Radboud University Medical Center, 6525 GA Nijmegen, The Netherlands; 3 Department of Urology, Radboud University Medical Centre, 6525 GA Nijmegen, The Netherlands; peter.mulders@radboudumc.nl (P.F.A.M.); jack.schalken@radboudumc.nl (J.A.S.)

**Keywords:** prostate cancer, cancer biomarkers, liquid biopsies, sPSA

## Abstract

**Simple Summary:**

In prostate cancer diagnosis and prognosis, clinicians often must take decisions on which prostate cancer-suspected patients should undergo biopsy and which prostate cancer-diagnosed patients should undergo treatment, to overcome overdiagnosis and overtreatment. Many biomarker-based tests have been developed and commercialized in order to guide decision-making processes of clinicians. Since a variety of biomarker-based tests are currently available, this review provides an overview of all commercially available tests for prostate cancer diagnosis and prognosis. For the diagnostic setting, the PHI, 4K, MiPS, SelectMDx, ExoDx, Proclarix, ConfirmMDx, PCA3 and PCMT are discussed, while for the prognostic setting, the Prolaris, OncotypeDx and Decipher test are discussed. Following, an overview is provided of the studies available comparing the performance of biomarker tests. Next, the use of biomarker tests complementary to the use of multiparametric magnetic resonance imaging in diagnosis of prostate cancer is discussed, which is an upcoming tool in prostate cancer diagnosis.

**Abstract:**

In the diagnosis and prognosis of prostate cancer (PCa), the serum prostate-specific antigen test is widely used but is associated with low specificity. Therefore, blood-, urinary- and tissue-based biomarker tests have been developed, intended to be used in the diagnostic and prognostic setting of PCa. This review provides an overview of commercially available biomarker tests developed to be used in several clinical stages of PCa management. In the diagnostic setting, the following tests can help selecting the right patients for initial and/or repeat biopsy: PHI, 4K, MiPS, SelectMDx, ExoDx, Proclarix, ConfirmMDx, PCA3 and PCMT. In the prognostic setting, the Prolaris, OncotypeDx and Decipher test can help in risk-stratification of patients regarding treatment decisions. Following, an overview is provided of the studies available comparing the performance of biomarker tests. However, only a small number of recently published head-to-head comparison studies are available. In contrast, recent research has focused on the use of biomarker tests in relation to the (complementary) use of multiparametric magnetic resonance imaging in PCa diagnosis.

## 1. Introduction

Prostate cancer (PCa) is one of the most frequently diagnosed cancers in men. Estimates for 2018 predicted 1.28 million new diagnosed PCa cases, associated with over 358,000 deaths [1]. The serum prostate-specific antigen (sPSA) test is widely used in multiple clinical phases of disease; PCa detection, risk stratification and monitoring [2]. However, due to the poor specificity of the sPSA test, especially in patients within sPSA range 3–10 ng/mL, several new molecular biomarker tests have been developed, aiming to improve diagnosis and prognosis of PCa. Blood-, urinary- and tissue-based biomarker tests have been developed to be used in several clinical stages of PCa management. In the diagnostic setting, available tests predict presence of (high-grade) PCa and therefore can help clinical decision-making i.e., in deciding which patients should undergo a first biopsy or a repeat biopsy after a negative first biopsy. In the prognostic setting, tests can enable clinicians to improve risk-stratification of post-positive biopsy patients to distinguish patients requiring treatment and those who can be monitored under active surveillance. Moreover, in a post-radical treatment setting, biomarker panels can be a valuable tool to determine those patients who benefit from secondary treatment. There are many biomarkers under study but, in this review, only the commercially available biomarker tests intended to be used in diagnostic and prognostic settings in the field of PCa will be discussed.

## 2. Biomarker Tests

### 2.1. Pre-Biopsy: Who to Biopsy?

#### 2.1.1. PHI

The Prostate Health Index (PHI; Beckman Coulter Inc., Brea, CA, USA) is a blood-based test designed to determine risk of PCa in men (age ≥ 50) with sPSA levels in the “gray zone” (4–10 ng/mL) and non-suspicious digital rectal exam (DRE) findings. The PHI score, providing probability of PCa on biopsy, can help to identify patients who are recommended for biopsy in both initial and repeat biopsy stages. The PHI test is based on three biomarkers: total PSA (tPSA), free PSA (fPSA) (the unbound form of PSA) and (-2) proPSA (p2PSA, an inactive isoform of PSA) [3]. The PHI test was initially validated by Catalona and co-workers in a large multicenter study including 892 men who underwent initial or repeat biopsy with normal DRE findings and sPSA between 2–10 ng/mL. They found that the PHI test could predict overall PCa (Area under the curve (AUC) = 0.70). At a sensitivity of 90%, the test reaches a specificity of 26.2% (PHI cutoff value 24.1) [4]. Results correspond with a large validation study (*N* = 1362, sPSA 1.6–8.0 ng/mL) in which PHI showed an AUC of 0.74 (95% confidence interval (CI): 0.71–0.76) for predicting PCa upon biopsy [5]. Although the test only provides a result of the risk of overall PCa, several studies have been conducted evaluating PHI performance in predicting high-grade PCa (hg-PCa), defined as GS ≥ 7. During the initial validation study on men waiting for initial or repeat biopsy, the test showed an AUC of 0.72 for distinguishing PCa Gleason Score (GS) 4 + 3 and higher from GS ≤ 3 + 4 and no PCa [4]. Higher performance was observed in biopsy-naïve men: another large multicenter study of two independent cohorts showed PHI to be a predictor for PCa GS ≥ 7 (AUC = 0.78–0.82). When combining the cohorts, a 30% specificity was observed at a sensitivity of 95% (PHI cutoff: 22.9) [6]. Wang and co-workers performed a meta-analysis including 16 validation studies, resulting in a pooled AUC, sensitivity and specificity for overall PCa of 0.70 (0.65–0.74), 85% and 45%. The pooled AUC showed sensitivity and specificity for predicting hg-PCa were low: AUC = 0.67 (0.57–0.77), 90% and 17% [7]. It should be noted that the validation studies included in this meta-analysis used different PHI cutoff values.

The performance of PHI was also investigated in patients undergoing radical prostatectomy (RP), aiming to predict adverse final pathological outcomes. Multiple studies described that pre-operative PHI testing can accurately predict pathological outcomes (pT3 disease, pathologic GS ≥ 7 and GS upgrading) after RP and, therefore, PHI could have potential utility to predict which patients diagnosed with localized PCa might benefit from radical treatment and which patients benefit from active surveillance [8,9,10]; see Table 1.

Next to that, the performance of PHI in combination with other (clinical) parameters was investigated, such as the combination of PHI with miRNAs [30] and PHI combined with age, DRE result and prostate volume (PV) [31,32]. These studies showed increased performance of the PHI when combined with other biomarkers but require validation in larger cohorts.

#### 2.1.2. 4K Score

The four-kallikrein (4K) score (Opko Health Inc., Miami, FL, USA) is a blood-based test developed to identify hg-PCa in patients with suspicious DRE outcome or an elevated sPSA level. The test measures the level of a 4K-panel, consisting of four kallikrein protein biomarkers (tPSA, fPSA, intact PSA and human kallikrein-related peptidase 2), and combines these parameters with clinical information on age, DRE outcome and prior negative biopsy status into an algorithm. The 4K score (range 0–100) provides an estimate of the patient’s risk of hg-PCa upon biopsy and can help in decision-making on the patients need for biopsy. Moreover, the test result provides a risk estimate on the patient’s probability of developing distant metastases within 10 years, based on sPSA score, age and the 4K-panel.

The predictive value of the 4K-panel included in the 4K score was assessed in multiple European retrospective studies that consistently showed the 4K-panel to be predictive for hg-PCa upon biopsy [33,34,35]. An initial prediction model was developed, containing the 4K-panel combined with DRE result and age and was modified by adding previous biopsy status to the contemporary 4K-test algorithm [36]. In a study evaluating the performance of the currently used 4K score in the US, 26 urology sites were included resulting in the inclusion of 1012 men scheduled for prostate biopsy [13]. The study evaluated the diagnostic performance for detecting hg-PCa showing an AUC of 0.82 (95% CI 0.79–0.85). Since only 8.1% of the study population were African American (AA) patients, the population was not representative for AA. Therefore, a validation study was conducted on a population with a higher percentage (56%) of AA-patients. Similar results were observed in this population, showing an AUC of 0.81 (95% CI 0.77–0.86) for the prediction of hg-PCa [14].

The other 4K score endpoint, 10-year probability of developing distant metastasis, was validated by Statin and co-workers [37]. They conducted a retrospective case-control study nested in a Swedish population-based cohort, in which the 4K-panel was measured from over 12,500 men (including 1423 PCa cases of which 235 developed PCa metastasis) that were followed over 15 years. Marker expression levels were combined in a prespecified model, including 4K panel and age. The model’s ability to predict distant metastasis within 10–20 years was examined. This research showed a strong association between sPSA and risk of 15–20-year metastasis in patients aged 50 or 60 years. Moreover, the 4K score significantly improved the prediction of metastasis as compared to sPSA levels alone in patients aged 50–60 [37].

Since prostate biopsies are associated with the risk of under sampling and therefore result in the risk of missing PCa, the performance of biomarker tests was also evaluated in predicting surgical specimen outcomes. Punnen and co-workers assessed the ability of the 4K score to predict adverse pathological outcomes and non-organ confined PCa in RP specimen. The 4K score was significantly higher in men with adverse pathology and a significantly higher median 4K score was shown in patients with non-organ confined PCa as compared to patients with organ confined PCa. However, when adding 4K score to a clinical prediction model including other clinical variables present after RP for the prediction of aggressive cancers upon RP specimen, the 4K score showed no added value in the prediction [38].

Haese and co-workers examined if the use of 4K score could be extended to a post-biopsy setting, focused on patients with biopsy GS 3 + 3 or 3 + 4 in order to increase prediction of adverse pathology at RP and biochemical recurrence (BCR). This could be useful in risk stratification of patients with GS 3 + 3 (decide whether to perform a confirmatory biopsy before AS is recommended) and in patients with GS 3 + 4 (decide on treatment or active surveillance). The 4K score was evaluated for these endpoints in a patient group (*N* = 2330) with majority of men diagnosed with low- and intermediate-risk cancer (biopsy GS 3 + 3 or 3 + 4). The ability of discriminating adverse pathology increased when adding the 4K score to a clinical model for both patients with GS 3 + 3 (AUC = 0.72) and GS 3 + 4 (AUC = 0.66) [39].

#### 2.1.3. Michigan Prostate Score

The Michigan Prostate Score (MiPS; Mlabs, Ann Arbor, MI, USA) combines sPSA with the mRNA expression of two PCa-specific urinary markers Prostate Cancer Antigen 3 (PCA3) and Transmembrane Serine Protease 2 (TMPRSS2)—ETS-related gene (ERG) gene fusion (T2-ERG) to predict the risk of PCa and PCa GS ≥ 7. PCA3, a long non-coding mRNA, is shown to be highly overexpressed in PCa tissue [40]. T2-ERG fusion gene, involving an aberrant fusion of the ERG gene with the TMPRSS2 gene is highly specific for PCa and is found in the approximately 50% of clinically localized PCa cases in Caucasian men [41]. These genes are previously shown to be detectable in post-DRE urine specimen and therefore can be a useful biomarker [40,42]. Combining urinary PCA3 and T2:ERG with sPSA is shown to improve specificity on the prediction of hg-PCa, compared to sPSA alone [19].

The MiPS test is intended to be used on first-catch, post-DRE urine from men with a suspicious sPSA level considering initial or repeat biopsy. The test score ranges from 0 to 100, reflecting the probability on finding any PCa and hg-PCa upon biopsy. These multivariable logistic regression models (predicting cancer risk and high-grade cancer risk) were validated in a cohort of 1225 men (initial and repeat biopsy) showing that the MiPS outperforms sPSA alone, with an AUC of 0.75 for predicting the presence of PCa and an AUC of 0.77 for the prediction of hg-PCa [20]. After this initial validation study in 2017, the regression-based prediction model was not further validated in other cohorts. Recently, the Mi-Prostate Score is renamed into MyProstateScore (MPS, LynxDx, Ann Arbor, MI, USA) test and its usefulness in clinical decision-making is currently under evaluation [22].

#### 2.1.4. ExoDx Prostate Test (IntelliScore)

The ExoDx Prostate Test (EPI; Exosome Diagnostics, Cambridge, MA, USA) is an urine-based biomarker test that provides a risk score (range 1–100) that predicts the presence of hg-PCa in men (aged 50 years or older, sPSA 2–10 ng/mL) scheduled for initial biopsy [17]. This test incorporates the EPI score, which reflects the mRNA expression levels of ERG and PCA3 (normalized for SAM pointed domain-containing ETS transcription factor (SPDEF) mRNA level) measured in isolated urinary exosomes. Exosomes are small vesicles that contain cellular materials from the originating cell they are secreted from. The potential of urinary exosomes as biomarker is mainly explained by the fact that they can be isolated from urine (non-invasive) and exosomal content is protected from degradation by proteases by its lipid bilayer, and therefore exosomes are a highly stable source for biomarker discovery in urine [23]. An EPI score > 15.6 indicates an increased risk on hg-PCa. The risk score is designed to be used in conjunction with sPSA, mpMRI results and other factors and can be useful in guiding decisions regarding performing a biopsy, aiming to reduce the number of unnecessary biopsies. In contrast to other urinary biomarker tests for Pca, the ExoDx Prostate Test does not require a DRE prior to urine collection [24].

In the developmental study of the test, Donovan and co-workers investigated the performance in a population of 195 men waiting for first biopsy (PSA 2–10 ng/mL). The assay showed to predict hg-PCa (AUC = 0.76; 95% CI 0.69–0.84) [24]. Following, the gene panel ERG, PCA3 and SPDEF was optimized, followed by validation in a cohort (*N* = 519) of men waiting for initial biopsy with sPSA level between 2–10. At a cut-off of 15.6, the gene panel showed a sensitivity and corresponding specificity of 91.89% and 33.96% and an AUC of 0.71 (95% CI; 0.66–0.75) for discriminating hg-PCa from GS 6 and no PCa. Combining the test with Standard of Care (SOC, including sPSA level, age, race and family history) the ExoDx test outperformed SOC alone, with AUCs of 0.73 (95% CI; 0.68–0.77) and 0.63 (95% CI; 0.58–0.68), respectively, in discriminating between hg-PCa from GS 6 PCa and no PCa. The gene panel also outperformed the PCPT risk calculator (AUC of 0.62 (95% CI; 0.57–0.67)) [17].

In 2018, a two-phase clinical utility study was started, aiming to validate the EPI performance in a second prospective cohort and to assess its clinical utility. Results from the performance assessment study (*N* = 503, sPSA levels between 2–10, age ≥ 50) showed similar results: at a cutoff value of 15.6, EPI score performed better (AUC = 0.70 (95% CI; 0.65–0.75) compared to standard of care alone (AUC = 0.62, 95%CI; 0.57–0.67). The combined model of EPI + SOC showed the highest AUC: 0.71 (95% CI; 0.66–0.76) [18]. The clinical utility study examined the impact of the EPI score on biopsy clinical decision-making, incorporating 72 urologists of which 68% reported that the EPI score influenced their biopsy decision-making process. Including EPI score in the decision-making process resulted in 30% more detection of hg-PCa compared to SOC alone [25]. Recently, the performance of the EPI test was evaluated in repeat biopsy setting (*N* = 229), showing that the EPI score outperformed European Randomized Study of Prostate Cancer (ERSPC)-risk calculator and sPSA in predicting hg-PCa in men with prior negative prostate biopsy, with a NPV of 92% at the cutoff value of 15.6 and an AUC of 0.66 (95% CI 0.55–0.78) [26].

#### 2.1.5. Proclarix

The most recently launched (Feb, 2020) commercially available pre-biopsy PCa biomarker test is the Proclarix test (Proteomedix, Schlieren, Switzerland). This CE-IVD marked test provides a risk score (ranging from 0 to 100) for the prediction of hg-PCa upon initial biopsy. It is a blood-based test intended to be used in men with prostate volume ≥ 35mL, no history of PCa, normal DRE result and elevated sPSA levels (2–10 ng/mL). This immunoassay-based ELISA test is performed on the same blood sample as the sPSA test, measuring the cancer-related glycoproteins thrombospondin-1 (THBS1) and cathepsin D (CTSD). These cancer-associated proteins were previously shown to be associated with the development of PCa [43,44]. The Proclarix test combines the quantification of these proteins with age, sPSA and % free PSA (%fPSA) to obtain a patient’s individual probability of detecting hg-PCa on biopsy.

Initial studies showed the clinical potential of THBS1 and CTSD in combination with %fPSA for (high-grade) PCa detection [45,46]. Based on these studies, Proclarix was developed. In the most recent study, the final prediction model (THBS1, CTSD, age, tPSA and %fPSA) was established and validated in a retrospective cohort of biobanked serum samples (*N* = 955), from patients with sPSA 2–10, PV ≥ 35 mL and normal DRE. The results showed that the Proclarix risk score outperformed %fPSA alone in predicting hg-PCa. At a sensitivity of 90%, the test shows a specificity of 43%, NPV of 95% and PPV of 25% (*N* = 955) [21]. Until now, no additional validation studies regarding the established prediction model were performed. Currently, clinical observational studies are being conducted.

#### 2.1.6. SelectMDx

The urinary molecular biomarker based SelectMDx (MDxHealth, Inc., Irvine, CA, USA) test is performed on post-DRE urine specimen from patients considered for initial biopsy, with increased sPSA levels in the “gray zone” (4–10 ng/mL). The assay consists of a prediction model that combines profiling of mRNA expression of Homeobox C6 (HOXC6) and Distal-Less Homeobox 1 (DLX1) (using Kallikrein Related Peptidase 3 (KLK3) as internal reference) with clinical risk factors age, sPSA density and DRE result. The resulting risk score designed for men with increased sPSA levels provides the patients likelihood of detecting any PCa and it provides the likelihood of detecting hg-PCa upon biopsy and can be used in clinical decision-making. An initial gene expression discovery study resulted in the identification of the gene panel HOXC6, Tudor Domain Containing 1 (TDRD1) and DLX1, which have been shown to have independent additional predictive value in a predictor model for hg-PCa [47]. Based on the identified genes by Leyten and co-workers, a prediction model for hg-PCa was developed by Van Neste and co-workers [15], including a gene panel with HOXC6 and DLX1 and clinical risk factors. Following, the first prospective multicenter validation study was performed on post-DRE urine from men (*N* = 386) waiting for initial or repeat biopsy, with sPSA ≥ 3 ng/mL, abnormal DRE or family history of PCa. The prediction model achieved an AUC of 0.86 (95% CI; 0.80–0.92) in predicting hg-PCa upon biopsy [15]. In a following multinational validation study on a cohort including men with sPSA < 10 ng/mL that were scheduled for initial biopsy, the performance was evaluated again. The prediction model outperformed PCPTRC risk calculator, with AUC’s, sensitivity, specificity and NPV of 0.82 (95% CI; 0.79–0.86), 89%, 53% and 95%, respectively, for SelectMDx, using a risk score cutoff of −2.8 [16].

### 2.2. Post-Negative Biopsy: Who to re-Biopsy?

#### 2.2.1. Progensa PCA3-Test

The Progensa PCA3-Test (Hologic Inc., Marlborough, MA, USA) is a post-DRE urinary biomarker test developed to predict the presence of PCa upon repeated biopsy. The PCA3 assay measures mRNA levels of PCA3 and sPSA, resulting in a risk score [48]. A PCA3 score < 25 is associated with lower risk on finding PCa upon biopsy. The score is intended to be used in combination with standard-of-care diagnostic parameters in men who underwent one or more previous negative biopsies and who are recommended for repeat biopsy [49]. The test is the first urinary biomarker test approved by the FDA (2012), approved for clinical indication of men aged ≥ 50 with increased sPSA levels and a history of negative prostate biopsy (cutoff 25).

In the initial validation study by Marks and co-workers [50], the test showed an AUC of 0.68 (95% CI 0.60–0.76) for distinguishing a positive from a negative repeat biopsy result. Multiple PCA3 cutoffs were evaluated; cutoffs of 10 and 35 showed a sensitivity of 87% and 58%, respectively, and a specificity of, respectively, 28% and 72%. At a cut-off value of 35, PCA3 outperformed sPSA [50]. In the following years, the diagnostic performance of the PCA3 test in decision-making on biopsy was well studied. Even though the FDA approval is provided for use in men that underwent one or more previous negative biopsies, many clinical studies were performed in men waiting on their first biopsy [51]. Ploussard and de la Taille reviewed clinical studies conducted on the performance of PCA3 in both initial and/or repeated biopsy settings. The observed AUC reported in studies including men in both initial and repeat biopsy setting, ranged from 0.64 to 0.83. In all included studies, PCA3 showed superiority over sPSA in terms of AUC [52]. Lee and co-workers examined the diagnostic accuracy of the PCA3 test for the diagnosis of PCa in initial and repeated biopsy setting. This most recently published meta-analysis included 54 studies. The PCA3 test had a pooled sensitivity of 0.71 (95% CI; 0.67–0.74) and a pooled specificity of 0.68 (95% CI; 0.63–0.74), including studies that used different cutoff values. The combined AUC was 0.75 (95% CI; 0.71–0.79). Sub-analysis showed no difference in performance between initial and repeated biopsy groups. It should be noted that the heterogeneity between included studies was indicated as high, partly caused by different cutoff values used (31 out of 54 studies used a cutoff value of 35) [53]. Similar results were found in a meta-analysis regarding AUC that included 46 studies, showing an AUC of 0.75 (95% CI 0.74–0.77). In contrast to the other meta-analysis, these results indicate a higher performance of the PCA3 test in the initial biopsy setting compared to repeated biopsy setting (for which the FDA approval is obtained). Moreover, the study provides evidence for selecting a cutoff of 35 as more appropriate in a clinical setting, instead of 25, which is FDA-approved [54]. This is also mentioned by Haese and co-workers and Deras and co-workers [55,56]. However, there still is conflicting reporting on the most suitable cutoff value [57].

#### 2.2.2. ConfirmMDx

ConfirmMDx (MDxHealth, Inc., Irvine, CA, USA) is a prostate biopsy Formalin-Fixed Paraffin-Embedded (FFPE) tissue-based epigenetic test that is used after initial negative biopsy to decide on repeat biopsy. The test is based on DNA methylation quantification of multiple tumor suppressor genes performed on histologically negative prostate tissue to detect an epigenetic field effect. Using Real Time Quantitative methylation-specific PCR (MSP), methylation of cytosines in promotor regions of the genes Glutathione S-Transferase Pi 1 (GSTP1), Adenomatous Polyposis Coli (APC) and Ras Association Domain Family Member 1 (RASSF1) is assessed, relative to the Beta-actin (ACTB) reference gene. The presence of epigenetic DNA methylation (positive of negative) of the biomarker panel is determined in up to 14 different areas on biopsy tissue specimen. The test result provides an overview of DNA methylation status of each of the three genes in all biopsied areas. Next to that, the EpiScore (reflecting normalized DNA methylation intensity) is combined in a prediction model with clinical factors age, sPSA, DRE and histopathology of the first biopsy to obtain a risk score that estimates of the likelihood of finding any PCa, low-grade PCa (GS ≤ 6) and hg-PCa (GS ≥ 7) upon repeat biopsy in methylation-positive men. The risk score was validated in a cohort of 803 men, reaching an AUC of 0.76 (95% CI; 0.68–0.84) for the prediction of GS ≥ 7 PCa upon repeat biopsy in methylation-positive men. Methylation status was shown to have an NPV of 96% [28]. The test result can guide decision-making for repeat biopsy after a negative result in initial biopsy and high sPSA and in identifying the prostate area that should be investigated during repeated biopsy.

In a clinical multicenter validation study (*N* = 320), the performance of the methylation status in predicting PCa was assessed. The epigenetic assay was shown to be a significant independent predictor of finding PCa upon repeat biopsy (OR: 2.69 (95%CI 1.6–4.51) and showed to have a NPV of 88% (95% CI; 85–91), with a sensitivity and specificity of 62% (52–72) and 64% (57–70) [58]. This corresponds with the findings of another validation study (*N* = 498), showing an NPV, sensitivity and specificity of 90% (95% CI; 87–93), 68% (57–77) and 64% (59–69) [59]. Recently, the clinical performance of the assay was assessed in a cohort of African American men undergoing repeat biopsy. This study reported no significant differences in sensitivity and specificity between this study and the previously described validation studies on predominantly Caucasian men [29]. A cutoff point to define a positive risk score in the prediction of hg-PCa is not published.

#### 2.2.3. Prostate Core Mitomic Test (PCMT)

The Prostate Core Mitomic Test (PCMT, MDNA Life Sciences Inc, West Palm Beach, FL, USA) is designed for patients with negative result on initial or repeated biopsy. The quantitative RT-PCR test, performed on prostate biopsy tissue, identifies a 3.4-kb deletion in mitochondrial DNA (mtDNA), which was previously shown to be elevated in PCa and normal-appearing cells adjacent to PCa [27]. In more detail: the test determines the presence of this mtDNA deletion in cells from histologically normal appearing prostate tissue, based on the ‘cancerization field effect’. Via this cancerization field effect, mutations in mtDNA can appear in cells in and around PCa cells and due to the high mutation rate in mtDNA, these mutations might be an early biomarker for the presence of malignant prostate cells [60]. The test outcome (positive or negative) with a cutoff of Ct 31 is used to stratify patients in the need for repeat biopsy. Robin son and co-workers (2010) assessed the clinical value of this test (*N* = 101), showing an AUC of 0.75 (0.63–0.87) for the prediction of re-biopsy outcome, associated with PCa missed in the first biopsy. The test shows an 84% sensitivity and a specificity of 54% for the detection of malignant cells in normal-appearing tissue, and a NPV of 92% [27]. However, only one validation study is performed to evaluate the performance of this test, containing a small number of cases (*N* = 101). This contributes to the wide 95% CI corresponding to the AUC.

### 2.3. Post-Positive Biopsy and Post-Definitive Treatment: Who to (Re)-Treat?

Three tests (OncotypeDx, Prolaris and Decipher) are commercially available for patients that have received a positive biopsy outcome in order to help in further clinical decision-making on active surveillance and treatment. Next to that, Decipher and Prolaris can also be used for men undergoing radical treatment in order to predict progression of the disease after RP, and can therefore be useful in decision-making on secondary treatment (Table 2).

#### 2.3.1. OncotypeDx

The OncotypeDx Genomic Prostate Score (Genomic Health, Redwood City, CA, USA), performed on prostate biopsy FFPE tissue, is used in patients diagnosed with National Comprehensive Cancer Network (NCCN) very-low-risk, low-risk or intermediate-risk PCa. The test consists of expression analysis of 17 genes (5 reference genes and 12 cancer associated genes) to deduce the Genomic Prostate Score (GPS). The GPS (scale 0–100) combined with clinical parameters (GS, sPSA, Clinical Stage, PV, PSA Density) indicates the patients individualized risk on three clinical endpoints: (1) finding hg-PCa (GS ≥ 4 + 3) and/or high-stage PCa (≥pT3) upon RP, (2) metastasis within 10 years and (3) PCa death within 10 years. These predictions on tumor aggressiveness and clinical risk upon RP can help in decision-making on whether a patient should go for active surveillance or treatment.

The first validation cohort (*N* = 395) showed that each 20-point increase in GPS score was associated with a 2.3-fold increased risk of hg-PCa (OR: 2.3 (95% CI 1.5–3.7)) and a 1.9-fold increased risk of high-stage PCa (OR: 1.9 (95% CI 1.3–2.9)) [73]. This trend was also observed in a second validation study, which showed that the GPS score was a good, independent predictor for long-term outcomes time to metastasis (hazard ratio per 20-points GPS increase: 3.8 (95% CI; 1.13–12.60) [61] and PCa-death (hazard ratio per 20-points GPS increase: 3.23) [62]. A meta-analysis showed that adding the GPS score to risk classification systems increased the AUC for the prediction of adverse pathology for NCCN risk group from 0.64 to 0.70 and for Cancer of the Prostate Risk Assessment (CAPRA) risk classification from 0.68 to 0.73 [74].

#### 2.3.2. Decipher

The Decipher Prostate Cancer Test (GenomeDx Biosciences, San Diego, CA, USA) can be utilized during risk-stratification of patients diagnosed with localized PCa upon biopsy indicated for definitive treatment or active surveillance. Moreover, the test can be used in patient’s post-RP specimen to decide if a patient can be monitored after RP or if the patient should be considered for salvage or adjuvant radiotherapy. This prognostic test incorporates quantification of mRNA expression levels of 22 genes in FFPE-prostate biopsy tissue or RP tissue. No clinical parameters are included. The test returns the Decipher Score (ranged between 0 and 1), which corresponds to a genomic risk level (low, intermediate or high) [65]. In case the test is used in post-positive biopsy setting on biopsy tissue, the patient’s risk of finding hg-PCa at RP (primary Gleason grade 4 or 5), risk on metastasis within 5 years after RP and risk on PCa mortality within 10 years after RP are reported.

These risk assessments can be used to inform clinicians about the possible course of treatment after biopsy, i.e., if active surveillance is appropriate or if intensive treatment such as RP, radiation or hormone therapy is needed. In case the test is used in post-RP setting on RP tissue, risk on metastasis within 5 years after RP and risk on PCa mortality within 10 years after RP are returned, which can guide decisions on the need for additional treatment after RP.

The initial validation cohort (*N* = 545) showed an AUC of 0.75 (0.67–0.83) for the prediction of 5-year metastasis in patients who underwent RP [65]. Another validation study regarding the endpoint of 5-year metastasis after RP showed an AUC of 0.79 (95% CI; 0.68–0.87) in RP-tissue [75]. Karnes and co-workers validated the Decipher Score (previously called the “Genomic Score” or “Genomic Classifier”) score to predict PCa specific mortality within 10 years after RP, showing an AUC of 0.73 (95% CI; 0.67–0.78) [64]. These described validation studies were all performed on RP-tissue. However, the test can also be used in patient’s post-positive biopsy, using biopsy tissue. Limited number of validation studies were performed on prostate biopsy tissue. The Decipher test was initially developed to be performed on RP-specimen, which was validated in several studies. The correlation between RP specimen and prostate biopsy specimen is high, regarding Decipher Score [76,77]. Klein and co-workers validated the Decipher Score on biopsy tissue, showing that this score can predict 10-year metastatic PCa risk following RP at the time of biopsy: hazard ratio per 0.1 point increase in Decipher Score: 1.75 (95% CI 1.97–2.81) [67].

#### 2.3.3. Prolaris

The Prolaris prognostic tissue-based test (Myriad Genetics, Salt Lake City, UT, USA) is developed for patients with localized PCa in order to provide help on decision-making on who to treat post-positive biopsy and post-RP. This test can complement the commonly used PCa risk stratification systems D’Amigo and NCCN by providing an individualized risk assessment on the risk of progression of the disease in patients with low or intermediate disease. The NCCN-guidelines recommend this test in patients with low to intermediate risk disease and at least a 10-year life expectancy. The test is performed on FFPE material from biopsy tissue or RP tissue. The test is based on quantification of gene expression levels of 31 cell-cycle progression associated genes in relation to expression levels of 15 reference genes. This results in a patient individualized cell-cycle progression-score, named Prolaris Molecular Score (PMS: range between 0 and 10). The PMS is reflected by combining the score with the NCCN risk category of this patient, stating if the tumor is less, equal or more aggressive as compared to the average risk of patients of the same NCCN-risk group. Next to that, the PMS is combined with clinical variables in an algorithm, resulting in the patient’s risk on 10-year disease specific mortality when active surveillance is considered and the patient’s risk on metastasis within 10 years with definitive treatment. These last risk assessments are then combined with the NCCN-risk group and a defined threshold for active surveillance, to advice on further treatment decisions [78]. When performing the test on post RP tissue, risk of BCR is provided.

Multiple validation studies assessed the prognostic value of the PMS. Sommariva and co-workers (2016) conducted a meta-analysis including five validation studies on clinical endpoints PCa mortality and BCR after RP. A pooled hazard ratio of 2.42 was found in a univariate model and 2.08 in a multivariate model for the risk of PCa mortality. For the risk on BCR, a pooled HR was calculated on 1.88 for the univariate model and 1.63 for the multivariate model [79]. Regarding the validation of the PMS in the prediction of metastasis, hazard ratios were found ranging from 2.21 to 4.19 [69,72].

## 3. Comparing Biomarker Tests

In a response to the need for development of biomarkers or genomic panels for diagnosis and prognosis of PCa, many biomarker tests have been developed over the last decade. For clinicians it is useful to know which biomarker test is best to be used in different clinical settings [80]. Therefore, head-to-head comparison studies of the tests are required. However, only a limited number of head-to-head comparative studies were performed to compare these tests (Table 3).

Multiple studies were conducted to compare the PHI and PCA3 test [83,84,85,86,88]. Hendriks and co-workers described all studies published before 2017 comparing the performance of these two diagnostic tests in multiple settings. In both initial and repeat biopsy setting, contradictory results were found between studies comparing PHI and PCA3 tests, concluding that it remains undetermined which test is best in initial/repeat biopsy setting [89]. No new studies have been published recently on the comparison between PHI and PCA3 in predicting PCa/hg-PCa upon biopsy.

A head-to-head comparative study compared PHI and 4K test score in initial biopsy setting in 513 men with sPSA levels between 3 and 15 ng/mL. Both tests were shown to outperform sPSA in the prediction of PCa. Next, the authors showed no differences in performance between PHI and 4K in predicting overall PCa (with AUCs of 0.69 (4K) and 0.74 (PHI)) and in hg-PCa (with AUCs of 0.72 (4K) and 0.71 (PHI)). However, no DRE information was available in this study, which is a requirement for the 4K test [81].

Vedder and co-workers examined performance of PCA3 and 4K when added to the ERSPC risk calculator. Subgroup analyses on data retrieved from men with sPSA ≥ 3.0 ng/mL (*N* = 202) showed the 4K score outperforming the PCA3 test in predicting PCa (AUCs of 0.78 and 0.62, respectively). In contrast, the PCA3 test performed better compared to 4K in distinguishing PCa in the total study population on *N* = 708 with no sPSA limits (AUCs 0.63 and 0.56). In multivariate modeling, both models added value to the ERSPC risk calculator in predicting PCa, without significant differences in performance between PCA3 and 4K [82].

Regarding prognostic biomarker tests, Alam and co-workers compared the agreement between OncotypeDx, Prolaris and Decipher test results. The percentage agreement was 75% between Prolaris and OncotypeDx (*N* = 12), 66.7% between Prolaris and Decipher (*N* = 12) and 50% between Decipher and OncotypeDx (*N* = 2) [90]. No other publications comparing prognostic tests were found.

## 4. MRI and Biomarkers

Multiparametric magnetic resonance imaging (mpMRI) is an emerging imaging tool for the use in diagnosis, prognosis and monitoring of PCa [91,92,93]. The European Association of Urology guidelines on PCa recommend the use of mpMRI prior to performing prostate biopsy in biopsy-naïve patients suspicious for PCa [94]. A recent meta-analysis showed a pooled sensitivity of 0.91 (0.83–0.95) and a pooled specificity of 0.37 (0.29–0.46) at a mpMRI cut-off value of PIRADS ≥ 3 for predicting GS ≥ 7 PCa in a combined initial and repeated biopsy setting [95]. Despite the high sensitivity of mpMRI for detecting hg-PCa, mpMRI also comes with drawbacks i.e., low specificity, high-costs, the need for specialized, expensive equipment, low sensitivity of predicting the presence of extracapsular extension and the requirement for expertise. Considering the inherent intra- and interobserver variability in reading the MRI images, MRI cannot be classified as a biomarker. Yet, the integration of advanced imaging (mpMRI) and biomarkers is likely to be the way forward.

Recent studies on previously commercialized biomarker tests are often focusing on how these diagnostic and prognostic tests act in the context of mpMRI. Studies are performed on comparing biomarker tests with mpMRI, but also on in which approach they can complement with each other. Hendriks and co-workers [96] assessed the association between SelectMDx score and finding suspicious significant lesion on mpMRI in patients (*N* = 172) undergoing biopsy. The SelectMDx score was significantly higher in mpMRI-positive (suspicious significant lesion on mpMRI: PI-RADS 4 or 5) compared to mpMRI-negative men (PI-RADS 1, 2 or 3)

This was also observed for PCA3. Next, the SelectMDx score performed better than the PCA3 score in predicting outcome of suspicious of PCa on mpMRI (AUC = 0.83 (0.77–0.89) for SelectMDx, compared to 0.65 (0.57–0.74) for PCA3), suggesting the potential of SelectMDx test to be used to stratify patients for mpMRI [96]. The advantages of complementary use of biomarker tests and mpMRI in selecting patients for subsequent biopsy was also established by Falagario and co-workers, demonstrating that the best approach was to perform a 4K test first and, when elevated (>7.5%), perform mpMRI followed by a biopsy if mpMRI ≥ PI-RADS 3 [97]. Moreover, two recently published studies compared the combination of 4K with MRI in the prediction for hg-PCa [98,99]. Punnen and co-workers [98] showed that the combination of 4K with mpMRI provides a higher predictive performance for hg-PCa (AUC = 0.82; 95% CI; 0.75–0.89), compared to the 4K score (AUC = 0.70; 95% CI; 0.62–0.79) or mpMRI (AUC = 0.74; 95% CI; 0.66–0.81) alone. Similar results (a higher net benefit when combining 4K with MRI) were found by Marzouk and co-workers [99], who evaluated a sequential testing approach in which an intermediate result on the 4K test is followed up with mpMRI in decision-making on prostate biopsies. Using this complementary strategy, both unnecessary mpMRI and biopsies can be avoided.

## 5. Discussion

**Figure 1:**
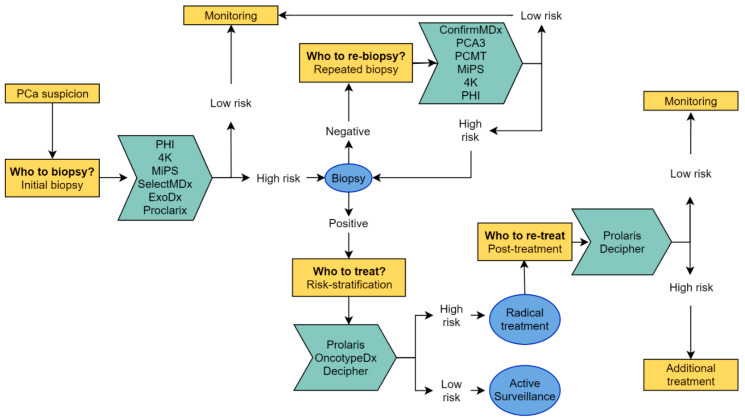
Schematic overview of commercialized prostate cancer biomarker tests intended to be used in various clinical settings.

In this review, commercially available biomarker tests that can be used in diagnostic and prognostic settings are reviewed (Figure 1). Next, head-to-head test biomarker comparison studies are described, as well as recent publications on the use of biomarker tests in the context of mpMRI as a diagnostic approach.

A clear gap exists between the number of new tests being marketed and the number of head-to-head studies comparing these biomarker tests. In more detail: only two head-to-head comparative studies have been published the last 5 years, for the in total over 10 different available tests. More head-to-head studies are required to indicate which tests are most suitable in which clinical phase. This is also described by the NCCN Guidelines for Prostate Cancer (2020), stating that no biomarker test can be recommended over any other due to small sample size and contradictory results in head-to-head comparison studies. Many studies were performed to validate the performance of the individual biomarker tests, which were varying in i.e., study population, biomarker model, biomarker cut-off value, clinical variables added to the model and analyzed endpoints Also, biomarker models have been compared in non-intended-use settings, which might result in misleading outcomes. Taken together, comparing tests performance in non-head-to-head studies can be problematic.

Moreover, the use of a cutoff value in order to indicate a test result as positive or negative must be argued. The optimal cutoff value depends on the indication and target population: a test developed for PCa screening in a non-suspicious population requires a highly specific test to prevent large number of false positives and so overdiagnosis, while a test intended to predict presence of hg-PCa in a population of PCa-suspicious men requires high sensitivity in order to reduce the risk on missing hg-PCa.

Next to the use of biomarker tests and mpMRI as diagnostic and prognostic tools, online risk prediction calculators play also an important role PCa risk-stratification. ERSPC Risk Calculator and the Prostate Cancer Prevention Trial Risk Calculator (PCPT-RC) are risk calculators that predict the individual’s risk on PCa and hg-PCa based on known clinical variable results [100,101,102]. These risk calculators have been compared to the 4K test showing no differences in performance [103,104]. Moreover, combining RPCRC and 4K score slightly reduced the number of unnecessary biopsies [104]. A minor improvement (increase in AUC of around 0.03) of predicting PCa was also observed when adding PCA3 or the 4K score to the ERSPC risk calculator [82].

Although many biomarker tests have been developed during the last ten years, efforts are still placed to improve diagnostic tools aiming to optimize clinical decisions. Recent research showed that the use of micro-RNAs, regulatory non-coding RNAs, circulating tumor cells and exosomes can be promising as biomarkers to differentiate indolent from aggressive PCa [105,106,107]. Other future directions seem to face an approach of combining blood-, urinary- and tissue-based markers with risk calculators in order to select patients for mpMRI and, subsequently, for biopsy.

## 6. Conclusions

In the last decade, many biomarker-based diagnostic and prognostic tests have become available on the market, intended to help clinicians in decision-making during PCa management. For both the pre-biopsy setting and the pre-treatment setting, a variety of commercial biomarker tests is available. A wide range of test-specific validation studies examined the performance of these biomarker tests, although the variation in quantity and quality of validation studies published between tests is considerable. Moreover, based on the currently available studies, it is hard to state which biomarker test is optimal in which setting. In order to compare the performance of these tests, more head-to-head validation studies directly comparing these tests are recommended. 

## Figures and Tables

**Table 1:** Commercialized biomarker tests for initial and repeated biopsy setting.

Test	Specimen	Setting (Pre-)	Biomarkers	Clinical Variables	Clinical Endpoint	AUC hg-PCa	Cutoff	Se	Sp	NPV	PPV	Ref
PHI	Blood	IBx, RBx	p2PSA, fPSA, tPSA	-	PCa	0.70–0.73 *	24.1–31.94	90–90.3	23–31.1	-	-	[3,4,11,12]
4K	Blood	IBx, RBx	tPSA, fPSA, iPSA, hK2	Age, DRE result, prior Bx-	hg-PCa, 10-y metastasis	0.81–0.82	-	-	-	-	-	[13,14]
SelectMDx	Post-DRE urine	IBx	DLX1, HOXC6, KLK3 mRNA, PSAd	Age, DRE result	PCa, hg-PCa	0.82–0.86	−2.8	89	53	95	34	[15,16]
ExoDx	Urine	IBx	PCA3, ERG, SPDEF ex-mRNA	-	hg-PCa	0.70–0.71	15.6	91.89–93	26.1–34	89.1–91.3	35.7–36.6	[17,18]
MiPS	Post-DRE urine	IBx, RBx	tPSA, PCA3, T2-ERG mRNA	-	PCa, hg-PCa	0.77	Not provided	92.6	33.4	92.6	33.2	[19,20]
Proclarix	Blood	IBx	THBS1, CTSD, tPSA, %fPSA	Age	hg-PCa	-	Not provided	90	43	95	25	[21]
PCA3	Post-DRE urine	RBx	PCA3, PSA mRNA	-	PCa	0.59–0.69 *	35	42–75.2	41.8–72	68–90.5	15.7–38.9	[17,18,22,23,24,25,26]
PCMT	PBx-tissue	RBx	mtDNA del	-	Presence mtDNA del	0.75	Ct: 31	84	54	91	-	[27]
ConfirmMDx	PBx-tissue	RBx	GSTP1, APC, RASSF1-hypm., PSA	Age, DRE result, histopath. IBx	PCa, hg-PCa, hyperm. site	0.76	Not provided	78	94.2	96	-	[28,29]

* AUC for PCa. Abbreviations: p2PSA: (-2) proPSA; 4K: four-kallikrein panel; AUC: area under the curve; Ct: Cycle threshold; CTSD: cathepsin D; DLX1: distal-less homeobox 1; DRE: digital rectal exam; ERG: ETS-related gene; ex-mRNA: exosomal mRNA; fPSA: free PSA; hg-PCa: high-grade PCa; hK2: human kallikrein-related peptidase 2; HOXC6: homeobox C6; hypm.: hypermethylation; IBx: initial Biopsy; iPSA: intact PSA; KLK3: kallikrein related peptidase 3; MiPS: Mi-Prostate Score; mRNA: messenger RNA; mtDNA del: mitochondrial DNA deletion; NPV: negative predictive value; PCa: prostate cancer; PCA3: PCa antigen 3; PCMT: Prostate Core Mitomic Test; PHI: Prostate Health Index; PPV: positive predictive value; prior Bx-: prior negative biopsy status; PSA: prostate-specific antigen; PSAd: PSA density; Ref: references; RBx: repeat Biopsy; Se: sensitivity; Sp: specificity; SPDEF: SAM pointed domain containing ETS transcription factor; T2-ERG: transmembrane protease serine 2-ERG; THBS1: thrombospondin-1; tPSA: total PSA.

**Table 2:** Commercialized biomarker for PCa risk-stratification.

Test	Specimen	Setting	Biomarkers	Clinical Variables	Clinical Endpoint (Risk on)	Performance	Ref.
OncotypeDx	FFPE tissue (from Bx)	Post-positive Bx: NCCN-low risk	17 genes	GS, PSA, Clinical Stage, PV, PSA Density	hg-PCa, high-stage PCa (≥pT3) upon RP, 10-y metastasis, 10-y PCa specific mortality	hg-PCa upon RP: OR/20 units GPS: 2.3 High-stage PCa upon RP: OR/20 units GPS: 1.9 10-y Metastasis: HR/20 units GPS: 3.8 HR/20 units GPS: 2.75 10-y PCa Mortality: HR/20 GPS units = 3.23	[61,62,63]
Decipher	FFPE tissue (from Bx or RP)	Post-positive Bx and post-RP	22 genes	No clinical parameters	Post-positive Bx - hg-PCa upon RP - 5-y metastasis - 10-y PCa specific mortality Post-RP - 5-y metastasis - 10-y PCa specific mortality	5-y Metastasis: Bx tissue: HR/0.1 point: 1.72 RP tissue: AUC range: 0.75–0.82 10-y PCa Mortality: RP tissue: AUC:0.73–0.78	[64,65,66,67]
Prolaris	FFPE tissue (from Bx or RP)	Post-positive Bx and post-RP	46 genes	Age at Bx, PSA, clinical T stage, percentage positive cores, GS, NCCN-risk category	Post-positive Bx - 10-y PCa specific mortality - 10-y metastasis Post-RP - BCR after RP	BCR: HR: 1.3–2.53 (multivar. model) 10-y PCa Mortality: HR: 1.7–2.6 (multivar. model) 10-y Metastasis: HR: 2.21–4.19 (multivar model)	[68,69,70,71,72]

Abbreviations: AUC: Area under the Curve; BCR: Biochemical recurrence; Bx: Biopsy; FFPE: Formalin-Fixed Paraffin-Embedded; GPS: Genomic Prostate Score (OncotypeDx); GS: Gleason Score; hg-PCa: High-Grade PCa; HR: Hazard Ratio; multivar: Multivariate; NCCN: National Comprehensive Cancer Network; OR: Odds Ratio; PSA: Prostate Specific Antigen; PV: Prostate Volume; Ref: references; RP: Radical Prostatectomy.

**Table 3:** Comparative head-to-head studies comparing biomarker tests for prostate cancer.

Compared Tests	Clinical Setting	Endpoint	AUC (95% CI)	Ref.
4K and PHI	IBx	PCa and hg-PCa	PCa 4K: 0.69 (0.65–0.73), PHI: 0.70 (0.66–0.75) hg-PCa 4K: 0.72 (0.67–0.77), PHI: 0.71 (0.66–0.76)	[81]
PCA3 and 4K	IBx	PCa	4K: 0.78 (0.69–0.85), PCA3: 0.62 (0.52–0.73)	[82]
PCA3 and PHI	IBx and RBx	PCa	PHI: 0.68 (0.62–0.74), PCA3: 0.74 (0.68 –0.79)	[83]
PCA3 and PHI	IBx and RBx	PCa	PHI: 0.70, PCA3: 0.59	[84]
PCA3 and PHI	IBx	PCa	PHI: 0.77 (0.72–0.83), PCA3: 0.73 (0.68–0.79)	[65]
PCA3 and PHI	IBx	PCa	PHI: 0.71 (0.61–0.80), PCA3: 0.66 (0.57–0.75)	[85]
PCA3 and PHI	IBx	PCa and hg-PCa	PCa PHI: 0.65, PCA3: 0.71 hg-PCa PHI: 0.80, PCA3: 0.55	[86]
PCA3 and PHI	IBx	PCa	PHI: 0.77, PCA3: 0.71	[87]

Abbreviations: 4K: four-kallikrein panel; AUC: area under the curve; hg: high-grade; IBx; Initial Biopsy; PCa: Prostate cancer; PCA3: Prostate Cancer Antigen 3; PHI: Prostate Health Index; Ref: references; RBx: Repeated biopsy.
